# Supplementary material for: Identification of SARS-CoV-2 Mpro inhibitors containing P1’ 4-fluorobenzothiazole moiety highly active against SARS-CoV-2
Source: Nat Commun. 2023 Feb 25;14:1076. doi: 10.1038/s41467-023-36729-0 (PMC9958325; doi:10.1038/s41467-023-36729-0)
Supplement: Supplementary file 1 — Supplementary information [file 41467_2023_36729_MOESM1_ESM.pdf]

Supplementary Information

Identification of SARS-CoV-2 M^{pro} Inhibitors Containing P1' 4- Fluorobenzothiazole Moiety Highly Active against SARS-CoV-2

Nobuyo Higashi-Kuwata[1], Kohei Tsuji[2], Hironori Hayashi[3], Haydar Bulut[4], Maki Kiso[5], Masaki Imai[5,6], Hiromi Ogata-Aoki[4], Takahiro Ishii[2], Takuya Kobayakawa[2], Kenta Nakano[7], Nobutoki Takamune[8], Naoki Kishimoto[8], Shin-ichiro Hattori[1], Debananda Das[4], Yukari Uemura[9], Yosuke Shimizu[9], Manabu Aoki[4], Kazuya Hasegawa[10], Satoshi Suzuki[11], Akie Nishiyama[11], Junji Saruwatari[12], Yukiko Shimizu[7], Yoshikazu Sukenaga[1], Yuki Takamatsu[1], Kiyoto Tsuchiya[13], Kenji Maeda[1], Kazuhisa Yoshimura[14], Shun Iida[15], Seiya Ozono[15], Tadaki Suzuki[15], Tadashi Okamura[7], Shogo Misumi[8], Yoshihiro Kawaoka[5,6,16], Hirokazu Tamamura[2], Hiroaki Mitsuya[1,6,17]*

[1]Department of Refractory Viral Diseases, National Center for Global Health and Medicine Research Institute, Tokyo, Japan

[2]Department of Medicinal Chemistry, Institute of Biomaterials and Bioengineering, Tokyo Medical and Dental University, Tokyo, Japan

[3]Department of Infectious Diseases, International Research Institute of Disaster Science, Tohoku University, Miyagi, Japan

[4]Experimental Retrovirology Section, HIV and AIDS Malignancy Branch, National Cancer Institute, NIH, Bethesda, MD, USA

[5]Division of Virology, Institute of Medical Science, University of Tokyo, Tokyo, Japan

[6]The Research Center for Global Viral Diseases, National Center for Global Health and Medicine Research Institute, Tokyo, Japan

[7]Department of Laboratory Animal Medicine, Research Institute, National Center for Global Health and Medicine, Tokyo, Japan

[8]Department of Environmental and Molecular Health Sciences, Faculty of Life Sciences, Kumamoto University, Kumamoto, Japan

[9]Center for Clinical Sciences, National Center for Global Health and Medicine, Tokyo, Japan

[10]Structural Biology Division, Japan Synchrotron Radiation Research Institute, Hyogo, Japan
[11]Department of Infectious Diseases, Tohoku University Graduate School of Medicine, Miyagi, Japan; [12]Division of Pharmacology and Therapeutics, Graduate School of Pharmaceutical Sciences, Kumamoto University, Kumamoto, Japan
[13]AIDS Clinical Center, National Center for Global Health and Medicine, Tokyo, Japan; [14]Tokyo Metropolitan Institute for Public Health, Tokyo, Japan
[15]Department of Pathology, National Institute of Infectious Diseases, Tokyo, Japan
[16]Influenza Research Institute, Department of Pathobiological Sciences, School of Veterinary Medicine, University of Wisconsin-Madison, Madison, WI, USA
[17]Kumamoto University Hospital, Kumamoto, Japan

***Contact information of the corresponding author:**
Hiroaki Mitsuya, M.D., Ph.D.
Email: hmitsuya@hosp.ncgm.go.jp

Contents:
Supplementary Table 1-4
Supplementary Figure 1-7
Supplementary Methods
References 2

Supplementary Table 1. Data collection and refinement statistics (molecular replacement)

	TKB245 (8DOX)	TKB248 (8DPR)
**Data collection**		
Space group	C 1 2 1	C 1 2 1
Cell dimensions		
a, *b*, *c* (Å)	115.33, 52.91, 45.60	115.40, 53.93, 45.68
α, β, γ (°)	90.00, 103.74, 90.00	90.00, 101.63, 90.00
Resolution (Å)	56.02 - 1.46 (1.512 - 1.46) *	56.52 - 2.004 (2.076 - 2.004) *
R_{sym} or R_{merge}	0.181 (10.74)	0.4678(13.6096)
$I / \sigma I$	8.68 (0.95)	9.16 (1.05)
Completeness (%)	99.0(93.1)	99.8(97.01)
Redundancy	8.5 (8.6)	8.60 (8.75)
**Refinement**		
Resolution (Å)	56.02 - 1.46	56.52 - 2.004
No. reflections	43216 (1907)	18591 (1842)
R_{work} / R_{free}	0.199/ 0.228	0.204 / 0.223
No. atoms	2505	2396
Protein	2337	2337
Ligand/ion	49	49
Water	119	10
B-factors	28.38	44.31
Protein	28.32	44.36
Ligand/ion	26.33	43.49
Water	30.47	35.89
R.m.s. deviations		
Bond lengths (Å)	0.015	0.012
Bond angles (°)	1.92	1.69

*Values in parentheses are for highest-resolution shell.

Supplementary Table 2. A list of SARS-CoV-2 variants used in this study

Virus Name	GISAID Accession ID	Pango lineage	Variant
hCoV-19/Japan/TY-WK-521/2020	EPI_ISL_408667	A	
hCoV-19/Japan/QHN001/2020	EPI_ISL_804007	B.1.1.7	VOC Alpha
hCoV-19/Japan/TY8-612-P0/2021	EPI_ISL_1123289	B.1.351	VOC Beta
hCoV-19/Japan/TY7-501/2021	EPI_ISL_833366	P.1	VOC Gamma
hCoV-19/Japan/TKYK01734/2021	EPI_ISL_2080609	B.1.617.2	VOC Delta
hCoV-19/Japan/TKYTK5356/2021	EPI_ISL_2378733	B.1.617.1	VOI Kappa
hCoV-19/Japan/TKYX00012/2021	EPI_ISL_8559478	BA.1.18	VOC Omicron
hCoV-19/Japan/TKYS02037/2022	EPI_ISL_9397331	BA.2.3	VOC Omicron
hCoV-19/Japan/TKYS14631/2022	EPI_ISL_12812500	BA.5.2.1	VOC Omicron
hCoV-19/Japan/NC928-2N/2021	EPI_ISL_7507055	BA.1.18	VOC Omicron
hCoV-19/USA/WI-UW-5250/2021	pending	B.1.617.2	VOC Delta
hCoV-19/Japan/UT-NCD1288-2N/2022	EPI_ISL_9595604	BA.2.10	VOC Omicron
hCoV-19/Japan/UT-NCD1757-1N/2022	EPI_ISL_14321746	BA.2.75	VOC Omicron

Supplementary Table 3. Species of M^{pro} or M^{pro}-compound complexes observed by native MS.

Species of M^{pro} or M^{pro}-compound complexes observed in native MS shown Figure 3a were identified by the comparison between each deconvoluted mass from measured spectra and corresponding theoretical mass.

Species	Theoretical mass [Da]	Experimental mass [Da][a]	Mass error
M^{pro}(Monomer)	33796.6	33796.5	-0.1
2xM^{pro}(Dimer)	67593.3	67597.6	4.3
2xM^{pro} + 1xTKB245	68246.9	68253.4	6.5
2xM^{pro} + 2xTKB245	68900.6	68912.3	11.7
2xM^{pro} + 1xTKB248	68263.0	68269.3	6.3
2xM^{pro} + 2xTKB248	68932.8	68929.2	-3.6
2xM^{pro} + 1xNirmatrelvir	68092.8	68086.5	-6.3
2xM^{pro} + 2xNIrmatrelvir	68592.3	68604.3	12

[a]Mean values of deconvoluted masses were determined using at least three charge states.

Supplementary Table 4. Species of glycine-added M^{pro} or glycine-added M^{pro}-compound complexes observed by native MS.

Species of glycine-added M^{pro} or glycine-added M^{pro}-compound complexes observed in native MS (Figure 3b) were identified by the comparison between each deconvoluted mass from measured spectra and corresponding theoretical mass.

Species	Theoretical mass [Da]	Experimental mass [Da][a]	Mass error
M^{pro}(Monomer)	33853.4	33853.5	0.1
2xM^{pro}(Dimer)	67706.8	67708.6	1.8
M^{pro} + 1xTKB245	34507.1	34519.4	12.3
2xM^{pro} + 1xTKB245	68360.5	68359.1	-1.4
2xM^{pro} + 2xTKB245	69014.2	69016.3	2.1
M^{pro} + TKB248	34523.2	34522.7	-0.5
2xM^{pro} + 1xTKB248	68376.5	68383.8	7.3
2xM^{pro} + 2xTKB248	69046.3	69052.5	6.2
M^{pro} + 1xNirmatrelvir	34352.9	34355.0	2.1
2xM^{pro} + 1xNirmatrelvir	68206.3	68213.8	7.5
2xM^{pro} + 2xNirmatrelvir	68705.9	68709.2	3.3

[a]Mean values of deconvoluted masses were determined using at least three charge states.

a

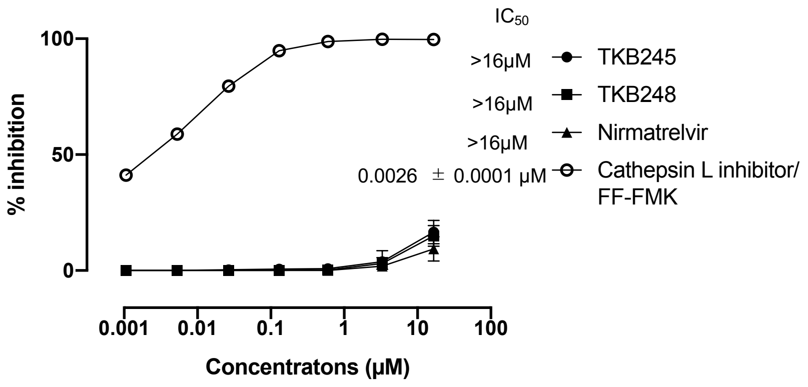

b

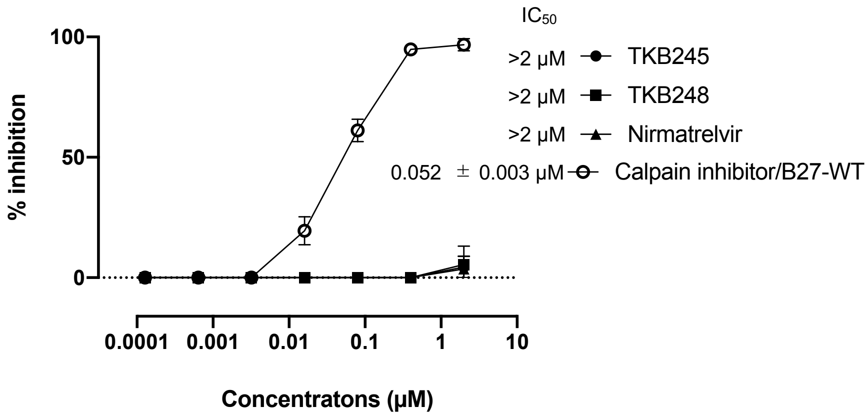

Supplementary Figure 1. Inhibition of human cysteine proteases, cathepsion L and calpains, by TKB245, TKB248, or nirmatrelvir. a. The inhibition curve of human cathepsin L by TKB245, TKB248, nirmatrelvir, or FF-FMK after a 30-minute incubation of the enzyme with increasing inhibitor concentrations. **b**. The inhibition curve of human calpains by TKB245, TKB248, nirmatrelvir, or B27-WT after a 60-minute incubation of the enzyme with increasing inhibitor concentrations. FF-FMK and B27-WT were examined as positive controls for human cathepsin L inhibitor and human calpain inhibitor, respectively. The data are shown with error bars in panels **a** and **b**. The mean IC_{50} values ± 1 S.D. of the assay results determined in triplicate (n=3). Source data are provided as a Source Data file.

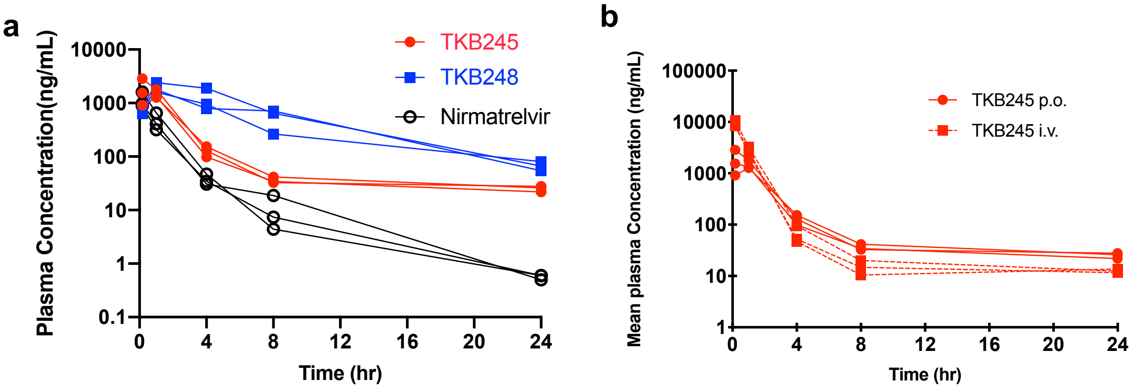

c

PK parameters	i.v.	p.o.
$t_{1/2}$(h)	1.25 ± 0.12	3.82 ± 0.48
AUC (μg/ml•hr)	10.7 ± 0.54	5.12 ± 0.43
CL (mL/hr/kg)	16.8 ± 0.85	35.2 ± 2.96
Vdss (mL/kg)	30.2 ± 3.81	194.1 ± 35.43
F [%]	47.7	

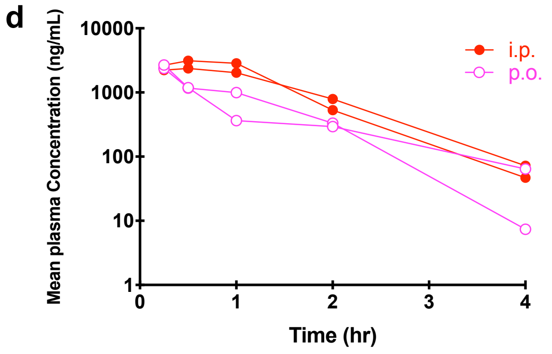

Supplementary Figure 2. Pharmacokinetic profiles of TKB245 and TKB248. a Human liver chimeric mice (PXB-mice) were treated with TKB245, TKB248, or nirmatrelvir (10mg/kg each) perorally (p.o.) and the time course of the plasma concentrations were examined (n=3 per each experimental group). **b** PXB-mice were intraperitoneally (i.p.) administered with TKB245 (10mg/kg) and the time course of plasma concentrations were compared those of p.o. (10mg/kg) (n=3 per each experimental group) **c** Summary of pharmacokinetic parameters of TKB245 in PXB-mice (n=3). AUC, area under the concentration-time curve; CL, clearance; F, oral bioavailability; t1/2, half-life; Vdss, distribution volume. Data are presented mean ± S.E. **d** ICR mice were administered with TKB245 (10 mg/kg) intraperitoneal (i.p.) or peroral (p.o.), and time course of the plasma concentrations was plotted. (n=2 per each experimental group). Source data are provided as a Source Data file.

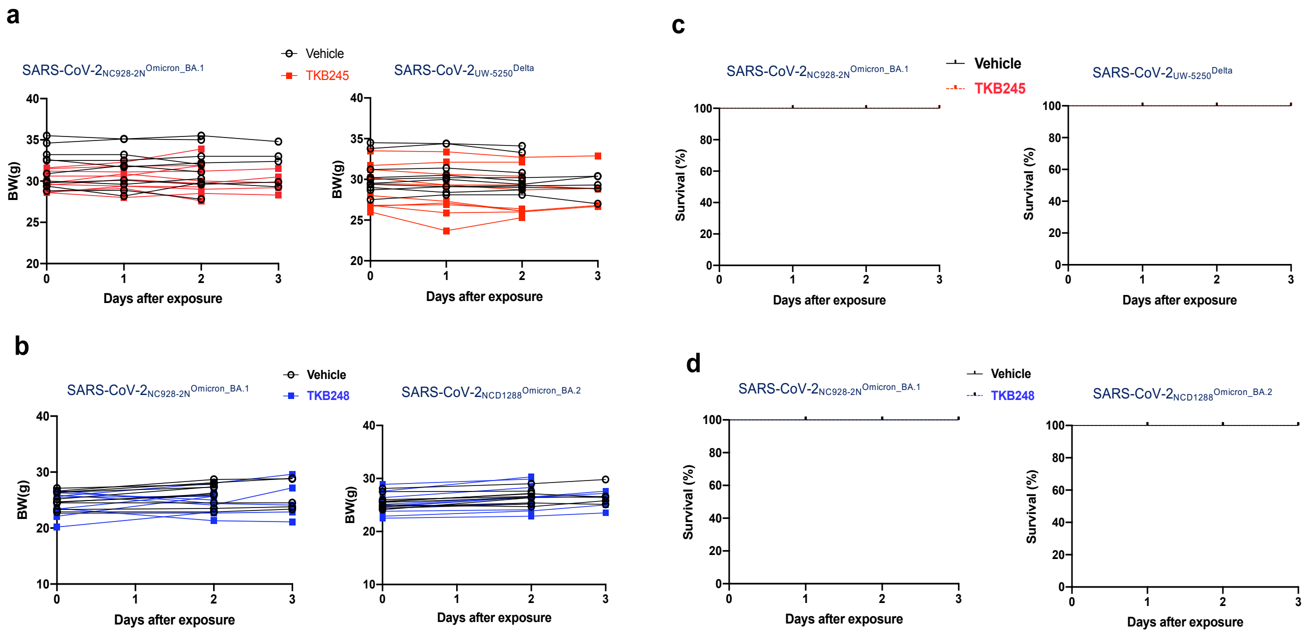

Supplementary Figure 3. The effect of TKB245 or TKB248 treatment on SARS-CoV-2 infected hACE2-knocked-in mice. Body weight of SARS-CoV-$2_{NC928\text{-}2N}{}^{Omicron\_BA.1}$ or SARS-CoV-$2_{UW\text{-}5250}{}^{Delta}$ infected and TKB245 or vehicle treated mice **(a)** and their survival rate **(c)** were monitored daily for 3 days post exposure, while body weight of SARS-CoV-$2_{NC928\text{-}2N}{}^{Omicron\_BA.1}$ or SARS-CoV-$2_{NCD1288}{}^{Omicron\_BA.2}$ infected and TKB248 or vehicle treated mice **(b)** and their survival rate **(d)** were monitored at day 2 and 3 post exposure. Data plots are individual body weight (n=5 per each experimental group). Survival data were depicted by Kaplan-Meier method. Source data are provided as a Source Data file.

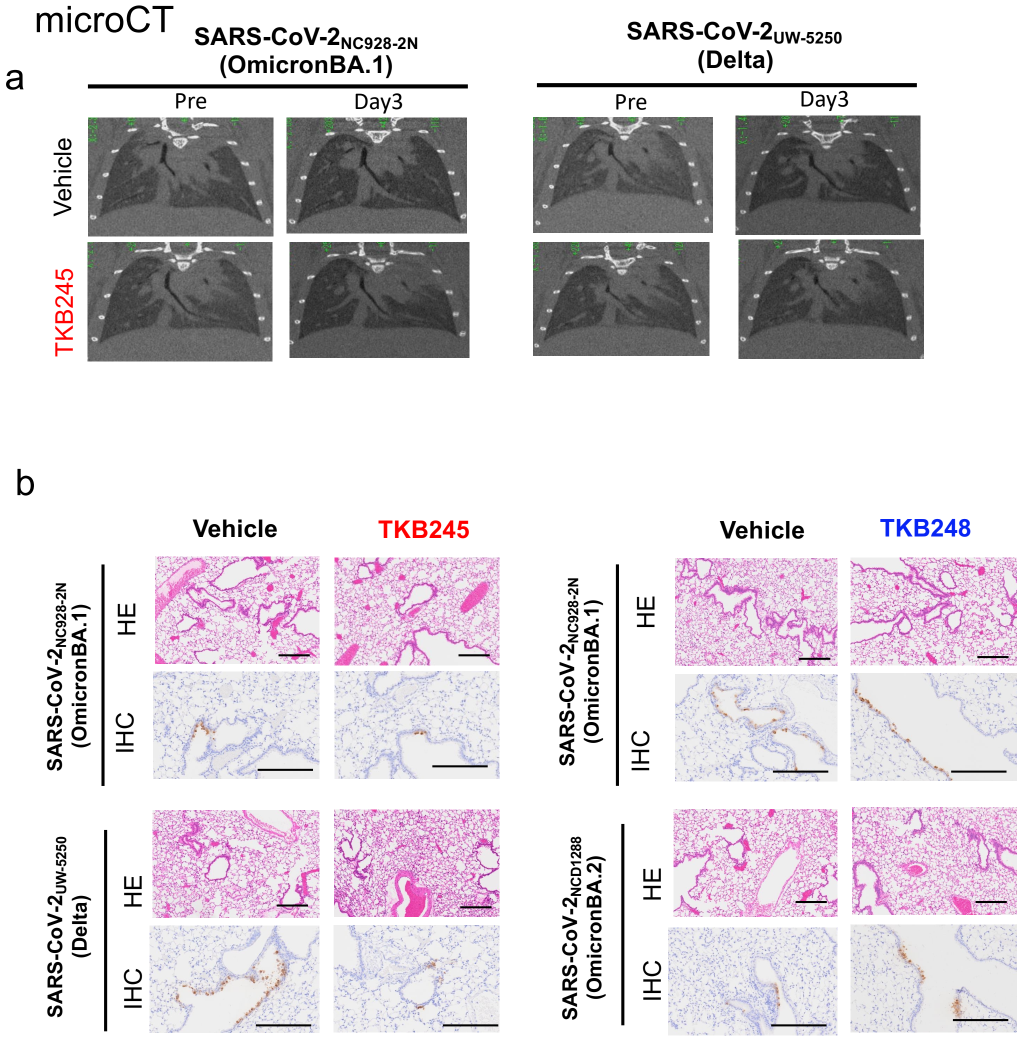

Supplementary Figure 4. Micro CT imaging and histopathological examination of SARS-CoV-2$_{\text{NC928-2N}}^{\text{Omicron BA.1}}$, SARS-CoV-2$_{\text{UW-5250}}^{\text{Delta}}$, or SARS-CoV-2$_{\text{NCD1288}}^{\text{Omicron BA.2}}$ infected and TKB245- or TKB248-treated hACE2-knocked-in mice.

a The chest CT images of the SARS-CoV-2$_{\text{NC928-2N}}^{\text{Omicron}}$ or SARS-CoV-2$_{\text{UW-5250}}^{\text{Delta}}$ -infected mice were captured using an *in vivo* micro-CT scanner before and on day 3 post infection under anesthesia as described previously[1,2]. No relevant differences were observed between vehicle control and TKB245 treatment group. **b** Histopathology examination were performed on excised lung tissues on day 3 post infection (n=3 per each experimental group) as described previously[1,2]. Representative images of the bronchi, and bronchioles and alveoli of mice are shown. Top row, haematoxylin and eosin (H&E)

staining. Bottom row, immunohistochemistry using a rabbit monoclonal antibody that detects SARS-CoV-2 nucleocapsid protein (1:1,000 dilution, catalog number 40143-R001, Sino Biological, Beijing, China). Scale bars, 200 μm. There was no inflammation in the lungs of both NC928-2N (Omicron, BA.1)-infected and UW-5250 (Delta)-infected mice regardless of TKB245 administration. In both Omicron, BA.1-infected and Delt-infected mice, nucleocapsid protein of SARS-CoV-2 was detected by immunohistochemistry in brown, however, there was no relevant change in number between the vehicle group and the TKB245 group. The same is true in lungs of TKB248 treated OmicronBA.1- and OmicronBA.2-infected mice.

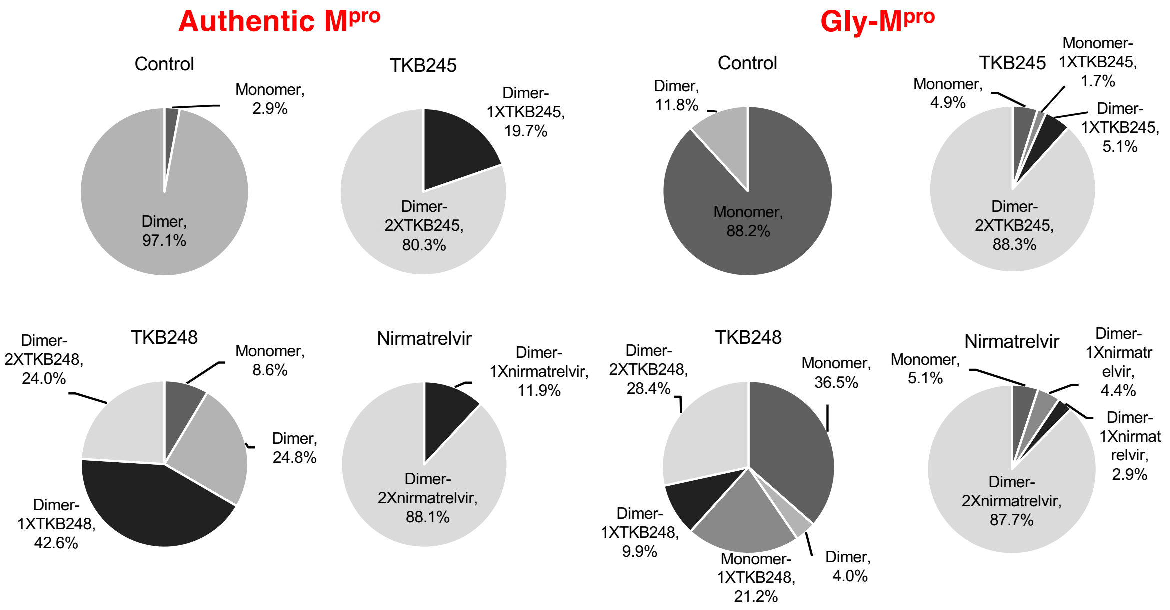

Supplementary Figure 5. Peak ratio of M^{pro} or M^{pro}-compound complexes observed by native MS. The peak ratio of the M^{pro} monomer, M^{pro} dimer or M^{pro}-compound complexes were calculated for each spectrum by integrating the relevant peak areas from all charge states.

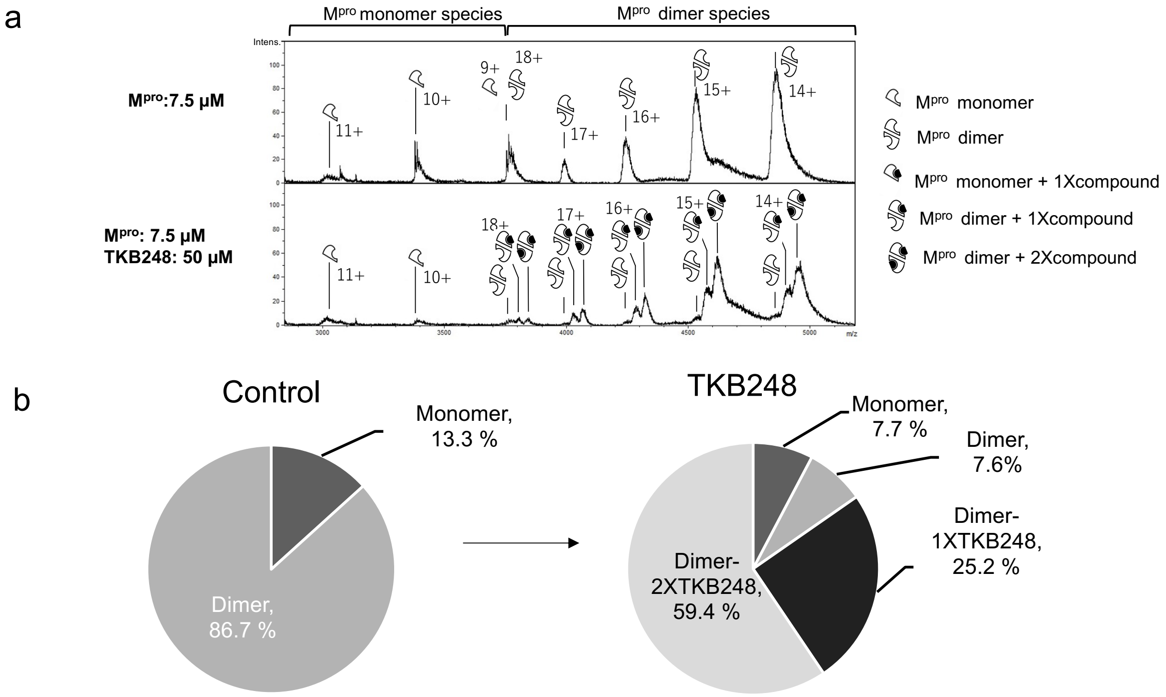

Supplementary Figure 6. Native mass spectrometric analysis of SARS-CoV-2 M^{pro}-inhibitor interaction. a. 7.5 µM of authentic M^{pro} was treated with 50 µM of TKB248. Relative native mass spectra of the M^{pro} with or without of TKB248 were shown. Charge states 9^+, 10^+ and 11^+ are annotated to mass spectra corresponding to M^{pro} monomer species and charge states 14^+, 15^+, 16^+, 17^+ and 18^+ are annotated to mass spectra corresponding to M^{pro} dimer species. **b.** Peak ratio of M^{pro} or M^{pro}-compound complexes observed by native MS. The peak ratio of the M^{pro} monomer, M^{pro} dimer or M^{pro}-TKB248 complexes were calculated for each spectrum by integrating the relevant peak areas from all charge states.

Supplementary Figure 7. Omitted electron densities (2Fo − Fc) of TKB245 (a) and TKB248 (b) including C145, contoured at the 1σ level.

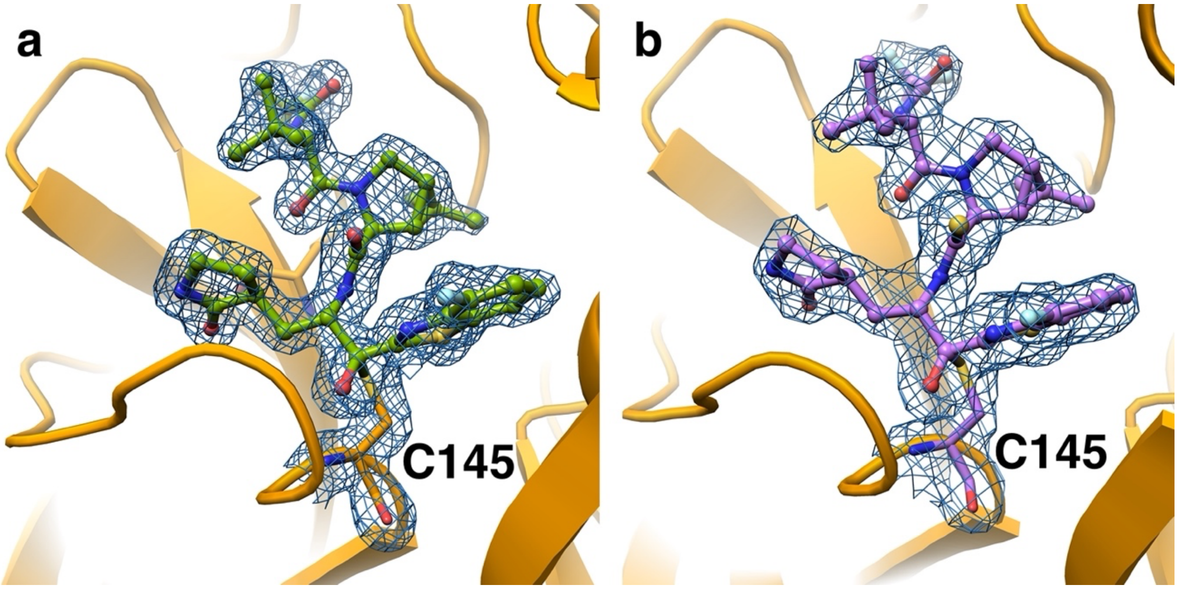

Supplementary Methods

General methods for synthesis and characterization of compounds.

I-I. General methods for synthesis

All reactions utilizing air- or moisture-sensitive reagents were performed in dried glassware under an atmosphere of nitrogen or argon (Ar), using commercially supplied solvents and reagents purchased from Sigma-Aldrich, Tokyo Chemical Industry Co., Ltd. (TCI), FUJIFILM Wako Pure Chemical Corporation, KANTO CHEMICAL CO.,INC., NACALAI TESQUE, INC., WATANABE CHEMICAL INDUSTRIES, LTD., KOKUSAN CHEMICAL Co.,Ltd., BLDpharm, Ambeed, Combi-Blocks, PharmaBlock Sciences (Nanjing), Inc., Enamine Ltd., 1ClickChemistry Inc., 1PlusChem, Chemspace LLC, CHEM-IMPEX INT'L INC., Matrix Scientific, and Absolute Chiral without further purification unless otherwise noted. Thin-layer chromatography (TLC) was performed on Merck $60F_{254}$ precoated silica gel plates and was visualized by fluorescence quenching under UV light and by staining with phosphomolybdic acid, *p*-anisaldehyde, or ninhydrin, respectively. Flash column chromatography was carried out with silica gel 60 N (Kanto Chemical Co., Inc.) or automatic silica gel flash column chromatography system (Isolera One (Biotage, Sweden) and Pure C-815 (Buchi, Switzerland)). Preparative RP-HPLC was performed using a Cosmosil $5C_{18}$-ARII column (20 × 250 mm, Nacalai Tesque, Inc., Japan) on a JASCO PU-2086 plus, PU-2087 plus, and PU-4086-Binary (JASCO Corporation, Ltd., Japan) in a linear gradient of MeCN containing 0.1% TFA (Solvent B) in H_2O containing 0.1% (v/v) TFA (Solvent A) at a flow rate of 10 cm^3 min^{-1}, and eluting products were detected by UV at 220 nm using JASCO UV-2075 plus and UV-4075 (JASCO Corporation, Ltd., Japan). For NP-HPLC, a CHIRALPAK IC semi-preparative column (10 x 250 mm, Daicel Corporation, Japan) were used on a JASCO PU-2086 plus in a linear gradient of isopropanol in *n*-hexane at a flow rate of 3.0 cm^3 min^{-1}, and eluting products were detected by UV at 220 nm using JASCO UV-2075 plus.

I-II. Characterization methods

^{1}H NMR(400 MHz or 500 MHz) and ^{13}C NMR(100 MHz or 125 MHz) spectra were recorded using a Bruker AVANCE III 400 spectrometer, Bruker AVANCE 500 spectrometer (Bruker, USA), and JNM-ECA500 (JEOL, Japan). Coupling constants are reported in Hertz, and peak shifts are reported in d (ppm) relative to $CDCl_3$ (^{1}H 7.26 ppm, ^{13}C 77.16 ppm) or MeOD (^{1}H 3.31 ppm, ^{13}C 49.00 ppm). Low- and high-resolution mass spectra were recorded on a Bruker Daltonics micrOTOF focus in the positive and negative detection mode. For analytical HPLC, a Cosmosil $5C_{18}$-ARII column (4.6 × 250 mm, Nacalai Tesque, Inc.) was employed with a linear gradient of MeCN containing 0.1% (v/v) trifluoroacetic acid (TFA) (Solvent B) in H_2O containing 0.1% (v/v) TFA (Solvent A) at a flow rate of 1.0 cm^3 min^{-1} on a PU-2089 plus (JASCO Corporation, Ltd.), and eluting products were detected by UV at 220 nm using JASCO UV-2075 plus. For NP-HPLC, a CHIRALPAK IC analytical column (4.6 x 250 mm, Daicel Corporation) was employed with a linear gradient of isopropanol in *n*-hexane at a flow rate of 1.0 cm^3 min^{-1} on a PU-2089 plus (JASCO Corporation, Ltd.), and eluting products were detected by UV at 220 nm using JASCO UV-2075 plus.

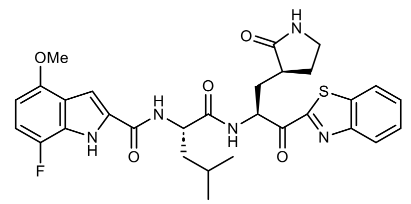

TKB125 (**1**)

***N*-((*S*)-1-(((*S*)-1-(Benzo[*d*]thiazol-2-yl)-1-oxo-3-((*S*)-2-oxopyrrolidin-3-yl)propan-2-yl)amino)-4-methyl-1-oxopentan-2-yl)-7-fluoro-4-methoxy-1*H*-indole-2-carboxamide (TKB125, 1).** ^{1}H NMR (500 MHz, $CDCl_3$) δ 9.99 (s, 1H), 8.66 (d, *J* = 6.3 Hz, 1H), 8.06-8.04 (m, 1H), 7.96-7.94 (m, 1H), 7.52-7.48 (m, 2H), 7.23 (s, 1H), 7.09-7.08 (m, 1H), 6.82-6.79 (m, 1H), 6.63 (brs, 1H), 6.28-6.26 (m, 1H), 5.74-5.70 (m, 1H), 4.99-4.95 (m, 1H), 3.87 (s, 3H), 3.35-3.34 (m, 2H), 2.71-2.65 (m, 1H), 2.52-2.51 (m, 1H), 2.30-2.24 (m, 1H), 2.19-2.14 (m, 1H), 2.06-1.98 (m, 1H), 1.85-1.74 (m, 2H), 1.70-1.64 (m, 1H), 0.97-0.94 (m, 6H); ^{13}C{^{1}H} NMR (125 MHz, $CDCl_3$) δ 192.1, 180.2, 172.9, 164.1, 161.0, 153.5, 150.3 (d, *J* =

2.0 Hz), 144.8 (d, J = 237.9 Hz), 137.3, 130.2, 128.0, 127.1, 126.2 (d, J = 15.9 Hz), 125.7, 122.5, 121.5 (d, J = 4.7 Hz), 108.8 (d, J = 17.9 Hz), 102.3, 98.7 (d, J = 6.1 Hz), 55.8, 55.3, 51.8, 42.5, 40.9, 39.3, 33.1, 28.5, 25.0, 23.0, 22.3; HRMS (ESI), *m/z* calcd for $C_{30}H_{33}FN_5O_5S$ $[M+H]^+$ 594.2181, found 594.2176; HPLC purity: >99% (Cosmosil 5C$_{18}$-ARII column 4.6 x 250 mm, 40 to 70% MeCN/H_2O containing 0.1% TFA over 30 min, 1.0 mL/min, 220 nm, room temperature, t_R = 19.2 min).

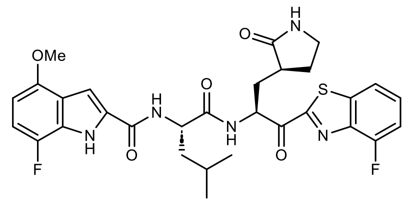

TKB198 (**2**)

7-Fluoro-*N*-((*S*)-1-(((*S*)-1-(4-fluorobenzo[*d*]thiazol-2-yl)-1-oxo-3-((*S*)-2-oxopyrrolidin-3-yl)propan-2-yl)amino)-4-methyl-1-oxopentan-2-yl)-4-methoxy-1*H*-indole-2-carboxamide (TKB198, 2). ^{1}H NMR (500 MHz, $CDCl_3$) δ 9.92 (s, 1H), 8.72-8.71 (m, 1H), 7.73-7.72 (m, 1H), 7.50-7.46 (m, 1H), 7.23-7.19 (m, 1H), 7.12-7.07 (m, 2H), 6.83-6.79 (m, 1H), 6.56 (s, 1H), 6.28-6.25 (m, 1H), 5.73-5.71 (m, 1H), 5.01-4.97 (m, 1H), 3.87 (s, 3H), 3.38-3.37 (m, 2H), 2.69 (s, 1H), 2.52 (s, 1H), 2.29-2.23 (m, 2H), 2.11-2.04 (m, 1H), 1.85-1.66 (m, 4H), 0.98 (d, J = 6.2 Hz, 3H), 0.97 (d, J = 6.2 Hz, 3H); $^{13}C\{^1H\}$ NMR (125 MHz, $CDCl_3$) δ 191.8, 180.1, 172.9, 164.6, 161.0, 157.3 (d, J = 260.46 Hz), 150.3 (d, J = 2.0 Hz), 144.8 (d, J = 237.7 Hz), 142.8 (d, J = 13.7 Hz), 139.8 (d, J = 2.2 Hz), 130.2, 129.0 (d, J = 6.9 Hz), 126.2 (d, J = 15.8 Hz), 121.5 (d, J = 5.7 Hz), 118.2 (d, J = 4.3 Hz), 112.5 (d, J = 17.6 Hz), 108.8 (d, J = 17.9 Hz), 102.3, 98.7 (d, J = 6.1 Hz), 55.8, 55.3, 51.7, 42.7, 40.9, 39.4, 32.9, 28.5, 25.0, 23.0, 22.3; HRMS (ESI), *m/z* calcd for $C_{30}H_{32}F_2N_5O_5S$ $[M+H]^+$ 612.2087, found 612.2090; HPLC purity: >99% (Cosmosil 5C$_{18}$-ARII column 4.6 x 250 mm, 40 to 70% MeCN/H_2O containing 0.1% TFA over 30 min, 1.0 mL/min, 220 nm, room temperature, t_R = 19.9 min).

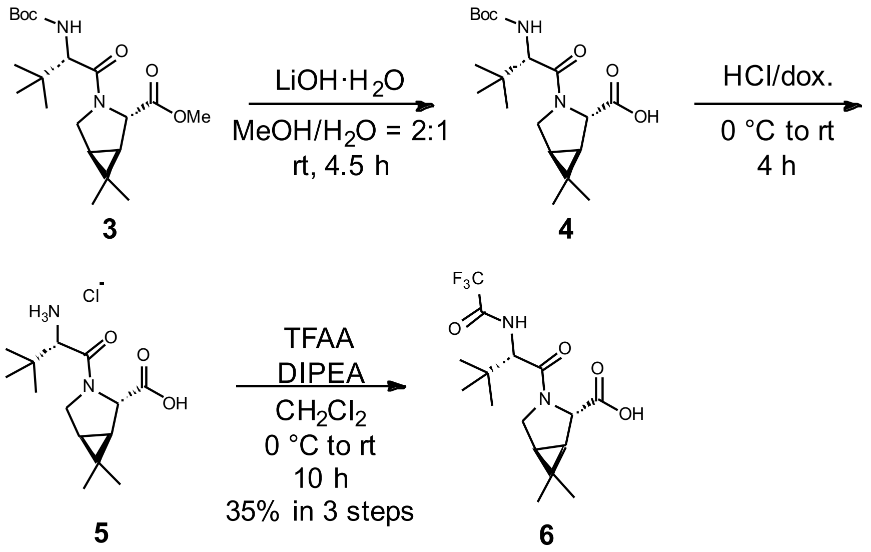

(1*R*,2*S*,5*S*)-3-((*S*)-3,3-Dimethyl-2-(2,2,2-trifluoroacetamido)butanoyl)-6,6-dimethyl-3-azabicyclo[3.1.0]hexane-2-carboxylic acid (6): To a solution of methyl ester **3** (705 mg, 1.84 mmol) in MeOH (12 mL)/H_2O (6.0 mL) was added LiOH·H_2O (116 mg, 2.76 mmol) and stirred at room temperature for 3 h. The mixture was added additional LiOH·H_2O (38.6 mg, 0.921 mmol) and stirred at room temperature for 1.5 h. The reaction mixture was cooled to 0 °C and acidified by the addition of 2 M HCl aq. The mixture was added brine and extracted with EtOAc. The organic layer was dried over $MgSO_4$ and concentrated under reduced pressure to obtain the corresponding carboxylic acid **4**, which was used immediately in next step without purification.

The crude carboxylic acid **4** (1.84 mmol) was treated with 4 M HCl in dioxane (dox., 9.2 mL) at 0 °C and the solution was stirred for 4 h at room temperature. The mixture was concentrated under reduced pressure to obtain the corresponding amine HCl salt **5**, which was used immediately in next step without purification.

To a solution of the crude amine HCl salt **5** (1.84 mmol) in CH_2Cl_2 (18 mL) was added *N*,*N*-diisopropylethylamine (DIPEA, 0.936 mL, 5.52 mmol) and trifluoroacetic anhydride (TFAA, 0.764 mL, 5.52 mmol) at 0 °C, and the mixture was stirred at 0 °C to room temperature for 10 h. The mixture was added H_2O and extracted with CH_2Cl_2. The organic layer was dried over $MgSO_4$ and concentrated under reduced pressure. The obtained crude compound was purified by silica gel flash column chromatography ($CHCl_3$/MeOH = 100:0 to 10:1) to afford the title compound **6** as a white solid (245 mg, 35% in 3 steps, rotational isomer mixture); ^1H NMR (500 MHz, $CDCl_3$) δ 7.49 (d, *J* = 9.7

Hz, 0.9H), 7.42 (d, *J* = 9.7 Hz, 0.1H), 6.43 (s, 1H), 4.61 (d, *J* = 9.7 Hz, 0.9H), 4.46-4.44 (m, 1H), 4.34 (d, *J* = 9.2 Hz, 0.1H), 3.93 (dd, *J* = 10.3 Hz and 5.2 Hz, 0.9H), 3.86 (d, *J* = 10.3 Hz, 0.9H), 3.77 (dd, *J* = 12.9 Hz and 5.4 Hz, 0.1H), 3.55 (d, *J* = 12.6 Hz, 0.1H), 1.68 (d, *J* = 7.4 Hz, 0.1H), 1.61 (d, *J* = 7.4 Hz, 0.9H), 1.54-1.47 (m, 1H), 1.08-0.98 (m, 12.3H), 0.89 (s, 2.7H); $^{13}C\{^1H\}$ NMR (126 MHz, $CDCl_3$) δ 175.3, 175.0, 169.6, 169.4, 157.4 (q, *J* = 37.6 Hz), 115.9 (q, *J* = 288.5 Hz), 61.1, 59.8, 58.4, 58.0, 48.3, 47.6, 36.9, 36.1, 32.8, 30.2, 27.4, 26.4, 26.4, 26.3, 26.2, 25.7, 20.2, 19.7, 13.3, 12.5; HRMS (ESI), *m/z* calcd for $C_{16}H_{22}F_3N_2O_4$ $[M-H]^-$ 363.1537, found 363.1534.

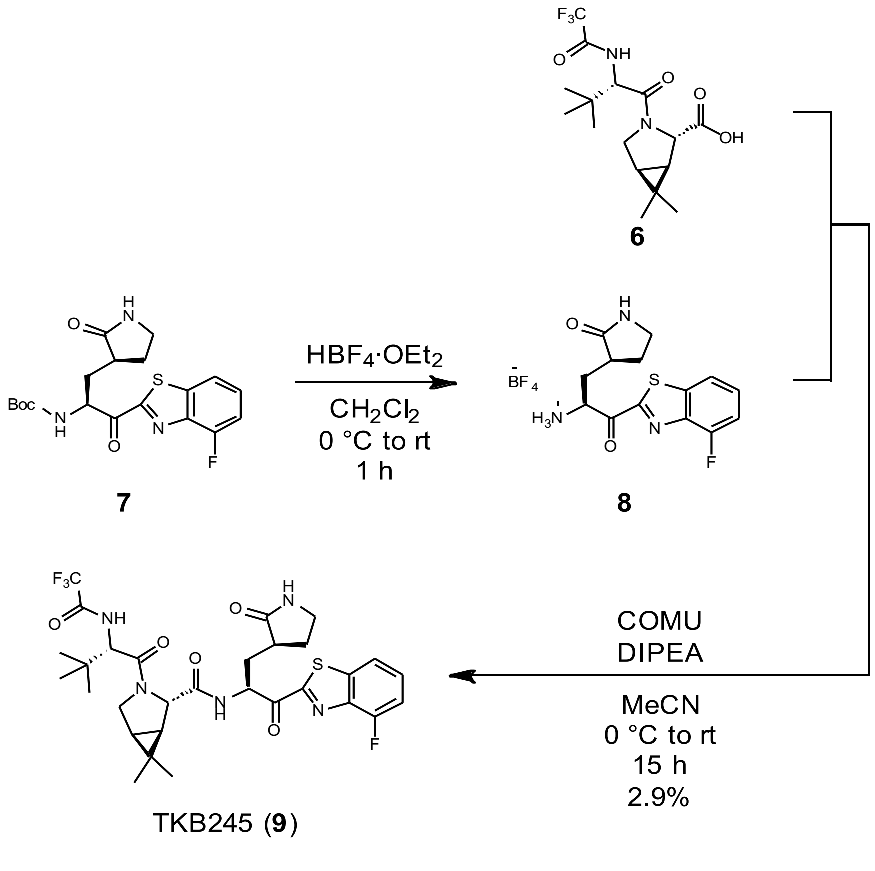

(1*R*,2*S*,5*S*)-3-((*S*)-3,3-Dimethyl-2-(2,2,2-trifluoroacetamido)butanoyl)-*N*-((*S*)-1-(4-fluorobenzo[*d*]thiazol-2-yl)-1-oxo-3-((*S*)-2-oxopyrrolidin-3-yl)propan-2-yl)-6,6-dimethyl-3-azabicyclo[3.1.0]hexane-2-carboxamide (TKB245, 9): To a solution of Boc protected amine **7** (143 mg, 0.350 mmol) in CH_2Cl_2 (3.5 mL) was added $HBF_4{\cdot}OEt_2$ (0.0961 mL, 0.700 mmol) dropwise at 0 ºC under argon, and the mixture was stirred at room temperature for 1 h. The reaction mixture was concentrated under reduced pressure, and the residue was washed with Et_2O containing 2% MeOH. The obtained crude amine HBF_4 salt **8** was used immediately in next step without purification.

To a solution of the crude amine **8** (0.180 mmol) in MeCN (1.5 mL) was added the carboxylic acid **6** (54.7 mg, 0.150 mmol), 1-[(1-(cyano-2-ethoxy-2-oxoethylideneaminooxy)dimethylaminomorpholino)]uronium hexafluorophosphate (COMU, 77.1 mg, 0.180 mmol), and DIPEA (0.0765 mL, 0.450 mmol) at 0 °C, and the mixture was allowed to stir for 15 h at room temperature. The reaction mixture was added saturated aqueous NH_4Cl and extracted with CH_2Cl_2. The organic layer was dried over $MgSO_4$ and concentrated under reduced pressure. The crude compound was roughly purified using automatic flash column chromatography system (Isolera One, $CHCl_3$/MeOH = 100:0 to 94:6) to obtain the crude title compound TKB245 (**9**). Further purification was performed by preparative RP-HPLC and CHIRALPAK IC semi-preparative NP-HPLC to afford the title compound TKB245 (**9**) as white powder (2.8 mg, 2.9%, rotational isomer mixture): ^{1}H NMR (400 MHz, $CDCl_3$) δ 8.91 (d, *J* = 5.0 Hz, 0.2H), 7.89 (d, *J* = 7.3 Hz, 0.8H), 7.76-7.71 (m, 1H), 7.54-7.46 (m, 1H), 7.28-7.22 (m, 1H), 7.09 (d, *J* = 9.2 Hz, 0.8H), 7.00 (d, *J* = 9.2 Hz, 0.2H), 6.55 (s, 0.8H), 6.32 (s, 0.2H), 5.88-5.82 (m, 0.8H), 5.64-5.59 (m, 0.2H), 4.57 (d, *J* = 9.4 Hz, 0.8H), 4.40 (s, 1H), 4.29 (d, *J* = 9.2 Hz, 0.2H), 3.98 (dd, *J* = 10.2 Hz and 5.3 Hz, 1H), 3.83-3.78 (m, 1H), 3.52-3.35 (m, 2H), 3.29-3.21 (m, 0.2H), 2.82-2.65 (m, 1H), 2.64-2.55 (m, 0.8H), 2.38-2.24 (m, 1H), 2.19-2.00 (m, 2H), 1.67 (d, *J* = 7.7 Hz, 0.2H), 1.60-1.58 (m, 0.8H), 1.55-1.52 (m, 0.8H), 1.47 (dd, *J* = 7.6 Hz and 5.5 Hz, 0.2H), 1.07-1.03 (m, 11H), 0.95 (s, 1.5H), 0.88 (s, 2.5H); ^{13}C{1H} NMR (101 MHz, $CDCl_3$) δ 192.2, 192.0, 181.9, 180.8, 171.2, 171.0, 169.2, 168.8, 164.4, 164.2, 157.4 (d, *J* = 261.1 Hz), 157.3 (d, *J* = 260.3 Hz), 157.1 (q, *J* = 37.4 Hz), 156.8-156.5 (m), 142.9 (d, *J* = 13.9 Hz), 142.8 (d, *J* = 14.0 Hz), 139.8 (d, *J* = 2.4 Hz), 129.2 (d, *J* = 7.3 Hz), 129.0 (d, *J* = 7.1 Hz), 118.3 (d, *J* = 4.5 Hz), 118.1 (d, *J* = 4.5 Hz), 116.0 (q, *J* = 288.0 Hz), 115.9 (q, *J* = 287.7 Hz), 112.7 (d, *J* = 17.5 Hz), 112.5 (d, *J* = 17.4 Hz), 62.0, 61.3, 58.3, 58.0, 56.0, 54.7, 48.7, 48.0, 41.3, 41.0, 39.7, 39.1, 37.2, 36.2, 33.8, 33.8, 32.5, 30.7, 29.3, 28.2, 27.9, 26.6,

26.5, 26.4, 26.2, 20.2, 19.5, 13.5, 12.7; HRMS (ESI), m/z calcd for $C_{30}H_{36}F_4N_5O_5S[M+H]^+$ 654.2368, found 654.2365; HPLC purity: >95% (Cosmosil $5C_{18}$-ARII column 4.6 x 250 mm, 40 to 70% $MeCN/H_2O$ containing 0.1% TFA over 30 min, 1.0 mL/min, 220 nm, room temperature, t_R = 23.2 min).

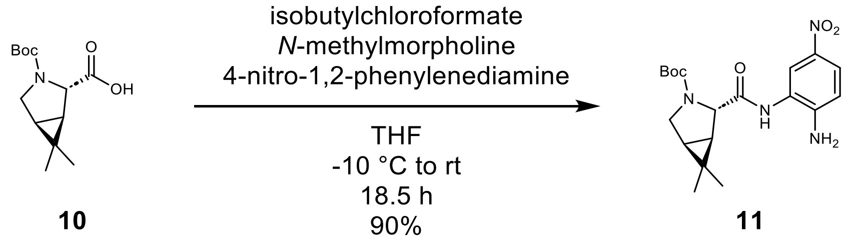

10 **11**

***tert*-Butyl (1*R*,2*S*,5*S*)-2-((2-amino-5-nitrophenyl)carbamoyl)-6,6-dimethyl-3-azabicyclo[3.1.0]hexane-3-carboxylate (11)**: To a solution of the compound **10** (638 mg, 2.50 mmol) in tetrahydrofuran (THF, 25 mL) was added *N*-methyl morpholine (0.550 mL, 5.00 mmol) and isobutyl chloroformate (0.361 mL, 2.75 mmol) at -10 °C under argon. After the reaction mixture was stirred for 10 min at -10 °C, 4-nitro-1,2-phenylenediamine (422 mg, 2.76 mmol) was added at -10 °C, and the mixture was stirred for 2 h at -10 °C, and then for 16.5 h at room temperature. The mixture was added sat. NH_4Cl aq. and extracted with EtOAc. The organic layer was dried over Mg_2SO_4 and concentrated *in vacuo*. The residue was purified using automatic flash column chromatography system (Isolera One, *n*-hexane/EtOAc = 23:2 to 3:2) to afford the title compound **11** as a yellow solid (881 mg, 90%, rotational isomer mixture): ^{1}H NMR (500 MHz, MeOD) δ 8.19 (d, J = 2.3 Hz, 0.4H), 8.03 (d, J = 2.3 Hz, 0.6H), 7.95 (dt, J = 9.2 Hz and 2.9 Hz, 1H), 7.92-7.85 (m, 1H), 6.84 (d, J = 8.6 Hz, 0.4H), 6.80-6.74 (m, 0.7H), 4.24 (s, 0.4H), 4.20 (s, 0.6H), 3.75 (dd, J = 11.2 Hz and 4.6 Hz, 1H), 3.44 (t, J = 10.9 Hz, 1H), 1.66-1.51 (m, 2H), 1.47 (s, 5.6H), 1.47 (s, 3.7H), 1.11 (s, 3H), 1.04 (s, 1H), 1.03 (s, 1.7H); ^{13}C{1H} NMR (126 MHz, $CDCl_3$) δ 170.5, 155.6, 147.4, 138.7, 123.8, 122.7, 121.4, 114.7, 81.6, 61.4, 47.3, 30.3, 28.6 (3C), 27.7, 26.3, 19.1, 12.7; HRMS (ESI), m/z calcd for $C_{19}H_{27}N_4O_5$ $[M+H]^+$ 391.1976, found 391.1972.

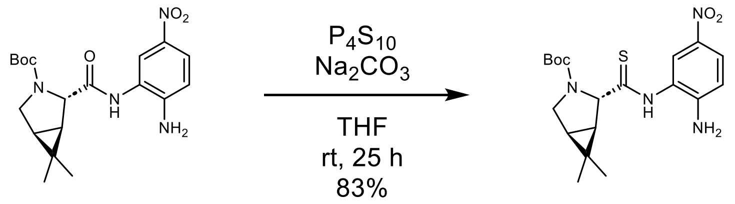

11 **12**

***tert*-Butyl (1*R*,2*S*,5*S*)-2-((2-amino-5-nitrophenyl)carbamothioyl)-6,6-dimethyl-3-azabicyclo[3.1.0]hexane-3-carboxylate (12)**: To a suspension of Na_2CO_3 (160 mg, 1.51 mmol) in THF (15 mL) was added phosphorus pentasulfide (335 mg, 1.51 mmol) at room temperature under argon. After 2.5 h stirring, the mixture became clear pale-yellow solution. The mixture was then added the compound **11** (586 mg, 1.50 mmol) and stirred for 25 h at room temperature. The mixture was concentrated under reduced pressure followed by flash column chromatography over silica gel with $CHCl_3$ (100%) to obtain the compound **12** as a yellow solid (83%, rotational isomer mixture): ^1H NMR (500 MHz, MeOD) δ 8.05-7.94 (m, 2H), 7.92-7.85 (m, 1H), 6.86 (d, *J* = 8.6 Hz, 0.3H), 6.78 (d, *J* = 9.2 Hz, 0.7H), 4.59 (s, 0.3H), 4.56 (s, 0.7H), 3.86 (dd, *J* = 10.9 Hz and 5.2 Hz, 1H), 3.49 (d, *J* = 10.9 Hz, 0.3H), 3.44 (d, *J* = 10.9 Hz, 0.7H), 1.66-1.49 (m, 2H), 1.46 (s, 2.5H), 1.45 (s, 6.6H), 1.11 (s, 3H), 1.06 (s, 1.7H), 1.05 (s, 0.9H); $^{13}C\{^1H\}$ NMR (126 MHz, $CDCl_3$) δ 205.6, 155.2, 148.9, 138.2, 125.8, 125.4, 122.0, 114.8, 81.7, 68.7, 47.7, 34.4, 28.7 (3C), 27.9, 26.3, 20.0, 13.1; HRMS (ESI), m/z calcd for $C_{19}H_{27}N_4O_4S$ $[M+H]^+$ 407.1748, found 407.1748.

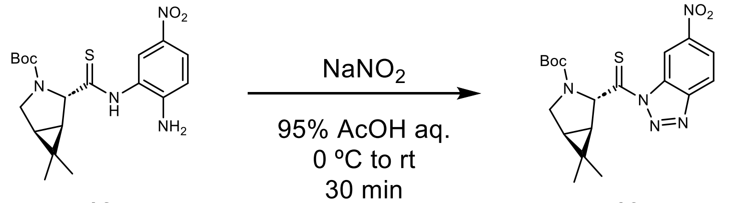

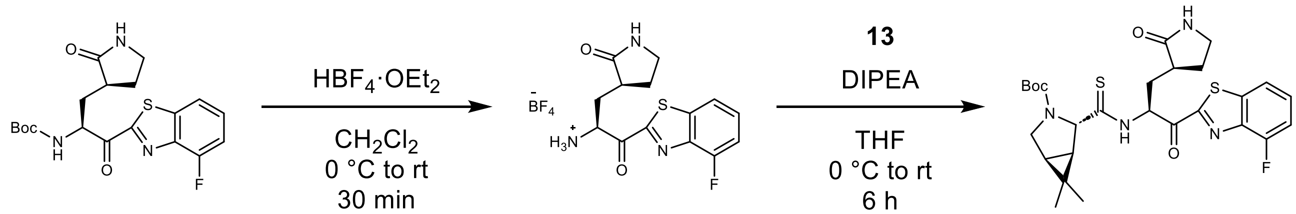

***tert*-Butyl (1*R*,2*S*,5*S*)-2-(((*S*)-1-(4-fluorobenzo[*d*]thiazol-2-yl)-1-oxo-3-((*S*)-2-oxopyrrolidin-3-yl)propan-2-yl)carbamothioyl)-6,6-dimethyl-3-azabicyclo[3.1.0]hexane-3-carboxylate (14)**: To a solution of compound **12** (276 mg, 0.679 mmol) in 95% aqueous AcOH (v/v, 70 mL) was added $NaNO_2$ (69.9 mg, 1.01 mmol) portionwise during 5 min at 0 °C. The mixture was then stirred for 30 min at room temperature. The mixture was added ice and H_2O (~40 mL), and the precipitate was collected by filteration and washed with cold H_2O. The residue was dried under reduced pressure overnight to obtain the crude **13** as an orange solid, which was used in next step without further purification.

The Boc-protected amine **7** (206 mg, 0.506 mmol) in CH_2Cl_2 (5.0 mL) was treated with $HBF_4 \cdot OEt_2$ (0.240 mL, 1.75 mmol) at 0 °C. The mixture was stirred for 30 min at room temperature, and then concentrated *in vacuo*. The residue was washed with 2% MeOH in Et_2O to obatin the crude amine HBF_4 salt **8**, which was used in next step without further purification.

The amine HBF_4 salt **8** (0.506 mmol) in THF (5.0 mL) was added DIPEA (0.0850 mL, 0.500 mmol) at room temperature. The crude **13** (178 mg, 0.426 mmol) in THF (5.0 mL) and DIPEA (0.255 mL, 1.50 mmol) was added to the solution at 0 °C and the mixture was allowed to stir for 6 h at room temperature. The reaction mixture was concentrated under reduced pressure, and the residue was roughly purified using automatic flash column chromatography system (Isolera One, $CHCl_3$/MeOH = 100:0 to 19:1) to afford the crude

title compound **14**, which was used in next step without further purification. HRMS (ESI), m/z calcd for $C_{19}H_{27}N_4O_4S$ $[M+H]^+$ 561.2000, found 561.2000.

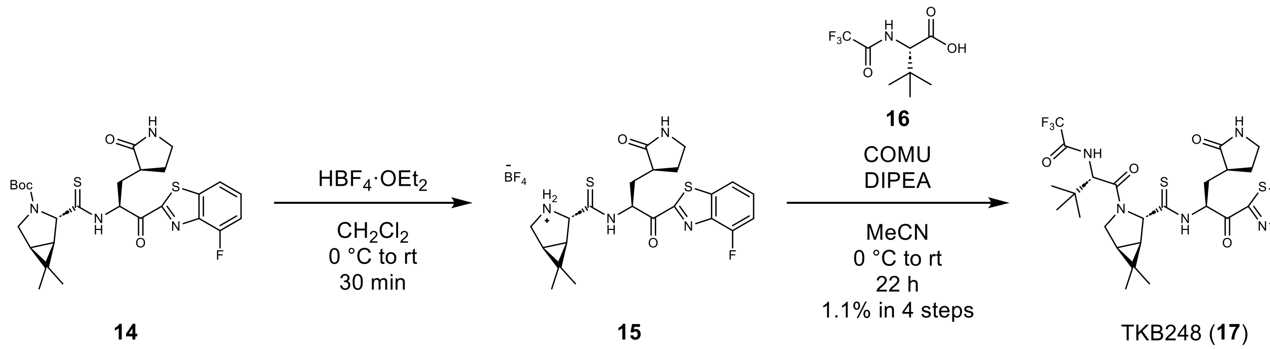

2,2,2-Trifluoro-*N*-((*S*)-1-((1*R*,2*S*,5*S*)-2-(((*S*)-1-(4-fluorobenzo[*d*]thiazol-2-yl)-1-oxo-3-((*S*)-2-oxopyrrolidin-3-yl)propan-2-yl)carbamothioyl)-6,6-dimethyl-3-azabicyclo[3.1.0]hexan-3-yl)-3,3-dimethyl-1-oxobutan-2-yl)acetamide (TKB248, 17): To a solution of the crude Boc protected amine **14** (0.426 mmol) in CH_2Cl_2 (4.0 mL) was added $HBF_4 \cdot OEt_2$ (0.210 mL, 1.53 mmol) dropwise at 0 ºC under argon, and the mixture was stirred at room temperature for 30 min. The reaction mixture was concentrated under reduced pressure, and the residue was washed by Et_2O containing 2% MeOH. The obtained crude amine HBF_4 salt **15** was used immediately in next step without purification.

To a solution of the crude amine **15** (0.426 mmol) in MeCN (2.0 mL) was added the carboxylic acid **16** (109 mg, 0.480 mmol) in MeCN (2.0 mL), COMU (206 mg, 0.481 mmol), and DIPEA (0.289 mL, 1.70 mmol) at 0 °C, and the solution was allowed to stir for 22 h at room temperature. The reaction mixture was added saturated aqueous $NaHCO_3$ and extracted with CH_2Cl_2. The organic layer was dried over $MgSO_4$ and concentrated under reduced pressure. The residue was roughly purified using automatic flash column chromatography system (Isolera One, $CHCl_3$/MeOH = 100:0 to 19:1) to afford the crude title compound TKB248 (**17**). Further purification was performed by preparative RP-HPLC and CHIRALPAK IC semi-preparative NP-HPLC to afford the title compound TKB248 (**17**) as a pale yellow powder (3.0 mg, 1.1% in 4 steps, rotational isomer mixture): ^{1}H NMR (500 MHz, $CDCl_3$) δ 7.75 (d, *J* = 8.0 Hz, 1H), 7.52-7.48 (m, 1H), 7.27-7.24 (m, 1H), 7.07-7.02 (m, 1H), 6.20 (brs, 0.3H), 6.16 (t, *J* = 4.9 Hz, 0.7H), 5.94 (s, 0.3H), 5.80 (s, 0.7H), 4.92 (s, 0.3H), 4.83 (s, 0.7H), 4.58 (d, *J* = 9.7 Hz, 0.7H), 4.42 (d, *J* = 9.7 Hz, 0.3H), 4.18 (dd, *J* = 10.3 Hz and 5.2 Hz, 0.7H), 4.07 (dd, *J* = 12.9 Hz and 6.0 Hz,

0.7H), 3.80-3.70 (m, 1H), 3.38-3.30 (m, 2H), 2.78-2.72 (m, 0.7H), 2.62-2.55 (m, 0.3H), 2.48-2.43 (m, 2H), 2.32-2.25 (m, 1H), 2.23-2.16 (m, 0.3H), 1.96-1.87 (m, 0.7H), 1.84-1.74 (m, 0.3H), 1.58-1.53 (m, 1H), 1.51-1.48 (m, 0.3H), 1.38-1.30 (m, 1H), 1.04-1.03 (m, 9H), 1.00 (s, 4.1H), 0.87 (s, 1.9H); $^{13}C\{1H\}$ NMR (125 MHz, $CDCl_3$) δ 203.0, 202.5, 190.5, 189.1, 181.0, 180.7, 169.6, 168.6, 165.6, 165.1, 157.4 (d, J = 258.8 Hz), 157.3 (d, J = 258.8 Hz), 157.4-155.9 (m), 142.9-142.6 (m), 139.8 (d, J = 2.4 Hz), 139.7 (br), 129.0-128.8 (m), 118.2 (d, J = 3.6 Hz), 118.1 (d, J = 3.6 Hz), 116.1 (q, J = 286.3 Hz), 116.0 (q, J = 286.3 Hz), 112.6 (d, J = 17.9 Hz), 112.5 (d, J = 17.9 Hz), 69.9, 67.2, 61.0, 59.6, 58.2, 58.1, 48.7, 48.5, 41.3, 41.2, 37.7, 37.7, 37.5, 37.0, 36.7, 33.6, 31.1, 30.5, 29.5, 29.1, 28.0, 26.7, 26.6, 26.3, 26.2, 25.4, 20.7, 19.8, 13.8, 13.0; HRMS (ESI), m/z calcd for $C_{30}H_{36}F_4N_5O_4S_2$ $[M+H]^+$ 670.2139, found 670.2141; HPLC purity: >99% (Cosmosil 5C_{18}-ARII column 4.6 x 250 mm, 50 to 90% MeCN/H_2O containing 0.1% TFA over 30 min, 1.0 mL/min, 220 nm, room temperature, t_R = 20.2 min).

References

1. Takamatsu Y, Imai M, Maeda K, Nakajima N, Higashi-Kuwata N, Iwatsuki-Horimoto K, Ito M, Kiso M, Maemura T, Takeda Y, Omata K, Suzuki T, Kawaoka Y, Mitsuya H. Highly Neutralizing COVID-19 Convalescent Plasmas Potently Block SARS-CoV-2 Replication and Pneumonia in Syrian Hamsters. *J Virol*. **96**, e0155121 (2022).
2. Uraki R, Kiso M, Iida S, Imai M, Takashita E, Kuroda M, Halfmann PJ, Loeber S, Maemura T, Yamayoshi S, Fujisaki S, Wang Z, Ito M, Ujie M, Iwatsuki-Horimoto K, Furusawa Y, Wright R, Chong Z, Ozono S, Yasuhara A, Ueki H, Sakai-Tagawa Y, Li R, Liu Y, Larson D, Koga M, Tsutsumi T, Adachi E, Saito M, Yamamoto S, Hagihara M, Mitamura K, Sato T, Hojo M, Hattori SI, Maeda K, Valdez R; IASO study team, Okuda M, Murakami J, Duong C, Godbole S, Douek DC, Maeda K, Watanabe S, Gordon A, Ohmagari N, Yotsuyanagi H, Diamond MS, Hasegawa H, Mitsuya H, Suzuki T, Kawaoka Y. Characterization and antiviral susceptibility of SARS-CoV-2 Omicron BA.2. *Nature* **607**, 119-127 (2022).
