# Supplementary material for: Identification of SARS-CoV-2 Mpro inhibitors containing P1’ 4-fluorobenzothiazole moiety highly active against SARS-CoV-2
Source: Nat Commun. 2023 Feb 25;14:1076. doi: 10.1038/s41467-023-36729-0 (PMC9958325; doi:10.1038/s41467-023-36729-0)
Supplement: Supplementary file 3 — Reporting Summary [file 41467_2023_36729_MOESM3_ESM.pdf]

nature portfolio

Corresponding author(s): Hiroaki Mitsuya

Last updated by author(s): Jan 31, 2023

Reporting Summary

Nature Portfolio wishes to improve the reproducibility of the work that we publish. This form provides structure for consistency and transparency in reporting. For further information on Nature Portfolio policies, see our Editorial Policies and the Editorial Policy Checklist.

Statistics

For all statistical analyses, confirm that the following items are present in the figure legend, table legend, main text, or Methods section.

n/a	Confirmed	
☐	☒	The exact sample size (*n*) for each experimental group/condition, given as a discrete number and unit of measurement
☐	☒	A statement on whether measurements were taken from distinct samples or whether the same sample was measured repeatedly
☐	☒	The statistical test(s) used AND whether they are one- or two-sided<br>*Only common tests should be described solely by name; describe more complex techniques in the Methods section.*
☒	☐	A description of all covariates tested
☐	☒	A description of any assumptions or corrections, such as tests of normality and adjustment for multiple comparisons
☐	☒	A full description of the statistical parameters including central tendency (e.g. means) or other basic estimates (e.g. regression coefficient) AND variation (e.g. standard deviation) or associated estimates of uncertainty (e.g. confidence intervals)
☐	☒	For null hypothesis testing, the test statistic (e.g. F, t, r) with confidence intervals, effect sizes, degrees of freedom and P value noted<br>*Give P values as exact values whenever suitable.*
☒	☐	For Bayesian analysis, information on the choice of priors and Markov chain Monte Carlo settings
☒	☐	For hierarchical and complex designs, identification of the appropriate level for tests and full reporting of outcomes
☒	☐	Estimates of effect sizes (e.g. Cohen's d, Pearson's r), indicating how they were calculated

Our web collection on statistics for biologists contains articles on many of the points above.

Software and code

Policy information about availability of computer code

Data collection	7500 Fast Real-Time PCR Instrument (ABI)<br>Cytation 5 cell imaging multi-mode reader (BioTek)<br>Agilent 6230B TOF LC/MS system (Agilent)<br>Inpact II ESI-QTOF mass spectrometer (Bruker)<br>SPring-8 BL41XU<br>MolRep, REFMAC5, PyMOL, UCSF Chimera
Data analysis	Prism 9.0 was used for the statistical analysis.

For manuscripts utilizing custom algorithms or software that are central to the research but not yet described in published literature, software must be made available to editors and reviewers. We strongly encourage code deposition in a community repository (e.g. GitHub). See the Nature Portfolio guidelines for submitting code & software for further information.

Data

Policy information about availability of data

All manuscripts must include a data availability statement. This statement should provide the following information, where applicable:

- Accession codes, unique identifiers, or web links for publicly available datasets
- A description of any restrictions on data availability
- For clinical datasets or third party data, please ensure that the statement adheres to our policy

All relevant data supporting the findings in this study are available within the paper in the Source Data file and the corresponding author upon reasonable request. Crystal structure data that support the findings of this study have been deposited in Protein Data Bank with the PDB IDs: 8DOX (https://www.rcsb.org/structure/8DOX) and 8DPR (https://www.rcsb.org/structure/8DPR).

Human research participants

Policy information about studies involving human research participants and Sex and Gender in Research.

Reporting on sex and gender	n/a
Population characteristics	n/a
Recruitment	n/a
Ethics oversight	n/a

Note that full information on the approval of the study protocol must also be provided in the manuscript.

Field-specific reporting

Please select the one below that is the best fit for your research. If you are not sure, read the appropriate sections before making your selection.

[x] Life sciences [ ] Behavioural & social sciences [ ] Ecological, evolutionary & environmental sciences

For a reference copy of the document with all sections, see nature.com/documents/nr-reporting-summary-flat.pdf

Life sciences study design

All studies must disclose on these points even when the disclosure is negative.

Sample size	No sample-size calculations were performed. No statistical methods were used to determine sample sizes. However, all sample sizes were chosen based on standard practices in the field and each experimental sample size in this study were described in the paper.
Data exclusions	No data exclusions.
Replication	All experiments with multiple biological replicates are indicated in the figure legends.
Randomization	Randomization for in vitro experiments was not required and was not performed because the aim of the experiments was to find more potent compounds than prototype or control compounds based on the structure and activity relationship. For in vivo animal experiments, randomization was adopted because such experiments required qualitative/quantitative evaluation to determine the efficacy of test compounds.
Blinding	Blinding for in vitro experiments was not required and was not performed because the aim of the experiments was to find more potent compounds than prototype or control compounds based on the structure and activity relationship. For in vivo animal experiments, blinding was adopted because such experiments required qualitative and quantitative evaluation of the efficacy of the compounds. Personnel who conducted the animal experiments were not aware of which compounds were being tested on the animals.

Reporting for specific materials, systems and methods

We require information from authors about some types of materials, experimental systems and methods used in many studies. Here, indicate whether each material, system or method listed is relevant to your study. If you are not sure if a list item applies to your research, read the appropriate section before selecting a response.

Materials & experimental systems

n/a	Involved in the study
☐	☒ Antibodies
☐	☒ Eukaryotic cell lines
☒	☐ Palaeontology and archaeology
☐	☒ Animals and other organisms
☒	☐ Clinical data
☒	☐ Dual use research of concern

Methods

n/a	Involved in the study
☒	☐ ChIP-seq
☒	☐ Flow cytometry
☒	☐ MRI-based neuroimaging

Antibodies

Antibodies used	COVID-19 convalescent plasma-derived IgG (ConvIgG) was used as a primary antibody (1/500 dilution)(IgG was purified at National Center for Global Health and Medicine) and Alexa Fluor® 488 AffiniPure Fab Fragment Goat Anti-Human IgG (H+L) as a secondary antibody (1/200 dilution)(Jackson ImmunoResearch, 109-547-003) for immunocytostaining. A rabbit monoclonal antibody that detects SARS-CoV-2 nucleocapsid protein (1:1,000 dilution, catalog number 40143-R001, Sino Biological, Beijing, China) was used for immunohistochemistry.
Validation	SARS-CoV-2 infection and IgG amounts were determined with RNA-qPCR and ELISA, respectively. ConvIgG was validated using immunostaining of SARS-CoV-2-infected and -uninfected VeroE6 cells and the data obtained were confirmed to be free from non-specific detection. The rabbit monoclonal antibody was validated using immunohistostaining of SARS-CoV-2-infected and -uninfected lung of hACE2 knock-in mice and the data obtained were confirmed to be free from non-specific detection.

Eukaryotic cell lines

Policy information about cell lines and Sex and Gender in Research

Cell line source(s)	African green monkey origin, Vero E6 cells from ATCC (CRL-1586). Hela-ACE2-TMPRSS2 cells were obtained from JCRB Cell Bank (JCRB1835), while A549-hACE2-TMPRSS2 cells were purchased from Invivo Gen (a549-hace2tpsa).
Authentication	The authentication was performed by morphology check under microscopes.
Mycoplasma contamination	All cell lines used in the present study were tested negative for mycoplasma contamination.
Commonly misidentified lines (See ICLAC register)	No commonly misidentified cell lines were used.

Animals and other research organisms

Policy information about studies involving animals; ARRIVE guidelines recommended for reporting animal research, and Sex and Gender in Research

Laboratory animals	Jcl:ICR (ICR) mice (5-6-week old) and PXB-mice (18-20-week old) were obtained from CLEA Japan (Tokyo, Japan) and PhoenixBio Co., Ltd.(Hiroshima, Japan), respectively. hACE2 knock-in mice(13-14-week old) , which were generated by inserting human ACE cDNA directly under the start codon in exon 2 of mouse Ace2 by the CRISPR/Cas9 system, were obtained from the RIKEN BioSource Center, Tukuba, Japan (official strain name: C.Cg-Ace2em1(ACE2)Okt : strain number RBRC11565).
Wild animals	No wild animals were used in the study.
Reporting on sex	In our preliminary experiments, both male and female mice were equally susceptible to infection with the SARS-CoV-2 strain used, and we observed comparable activity of the test compounds in the infected mice of either sex. Therefore, in our infection/treatment experiments reported in the current paper, we chose to use male mice in all experiments since sufficient numbers of mice were available at the time.
Field-collected samples	No field collected samples were used in the study.
Ethics oversight	All animal experiments were approved by the President of NCGM, the University of Tokyo and PhoenixBio Co., Ltd., following consideration by the Institutional Animal Care and Use Committee of NCGM (approval ID: no. 21057), the University of Tokyo (approval ID: PA19-72) and PhoenixBio Co., Ltd.(approval ID: 2722), and were carried out in accordance with institutional procedures, national guidelines and the relevant national laws on the protection of animals.

Note that full information on the approval of the study protocol must also be provided in the manuscript.
